# Osteogenic potential of heterogeneous and CD271-enriched mesenchymal stromal cells cultured on apatite-wollastonite 3D scaffolds

**DOI:** 10.1186/s42490-019-0015-y

**Published:** 2019-06-19

**Authors:** Sylvia Müller, Lyndsey Nicholson, Naif Al Harbi, Elena Mancuso, Elena Jones, Anne Dickinson, Xiao Nong Wang, Kenneth Dalgarno

**Affiliations:** 1 Institute of Cellular Medicine, Newcastle University, Newcastle upon Tyne, UK; 2 School of Engineering, Newcastle University, Newcastle upon Tyne, NE2 4HH UK; 3 Leeds Institute of Rheumatic and Musculoskeletal Medicine, University of Leeds, Leeds, UK

**Keywords:** Mesenchymal stromal cells, CD271 enrichment, Apatite-wollastonite glass ceramic 3D scaffold, Osteogenic regeneration

## Abstract

**Background:**

Mesenchymal stromal cells (MSCs) are widely used in clinical trials for bone repair and regeneration. Despite previous evidence showing a prominent osteogenic potential of 2D cultured CD271 enriched MSCs, the osteogenic potential of CD271 enriched cells cultured on 3D scaffold is unknown. Apatite-wollastonite glass ceramic (A-W) is an osteoconductive biomaterial shown to be compatible with MSCs. This is the first study comparing the attachment, growth kinetics, and osteogenic potential of two MSC populations, namely heterogeneous plastic adherence MSCs (PA-MSCs) and CD271-enriched MSCs (CD271-MSCs), when cultured on A-W 3D scaffold.

**Results:**

The paired MSC populations were assessed for their attachment, growth kinetics and ALP activity using confocal and scanning electron microscopy and the quantifications of DNA contents and p-nitrophenyl (pNP) production respectively. While the PA-MSCs and CD271-MSCs had similar expansion and tri-lineage differentiation capacity during standard 2D culture, they showed different proliferation kinetics when seeded on the A-W scaffolds. PA-MSCs displayed a well-spread attachment with more elongated morphology compared to CD271-MSCs, signifying a different level of interaction between the cell populations and the scaffold surface. Following scaffold seeding PA-MSCs fully integrated into the scaffold surface and showed a stronger propensity for osteogenic differentiation as indicated by higher ALP activity than CD271-MSCs. Furthermore, A-W scaffold seeded uncultured non-enriched bone marrow mononuclear cells also demonstrated a higher proliferation rate and greater ALP activity compared to their CD271-enriched counterpart.

**Conclusions:**

Our findings suggest that CD271-positive enrichment of a population is not beneficial for osteogenesis when the cells are seeded on A-W scaffold. Furthermore, unselected heterogeneous MSCs or BM-MNCs are more promising for A-W scaffold based bone regeneration. This leads to a conclusion of broader clinical relevance for tissue engineering: on the basis of our observations here the osteogenic potential observed in 2D cell culture should not be considered indicative of likely performance in a 3D scaffold based system, even when one of the cell populations is effectively a subset of the other.

## Background

Bone tissue regeneration is a complex process contributing to biological repair of bone defects which can develop in patients with a variety of complications. Mesenchymal stromal cells (MSCs) and bioactive scaffolds are increasingly used in translational research and clinical applications to enhance bone repair/regeneration [1, 2]. MSCs are multipotent progenitor cells with promising therapeutic potential for bone repair/regeneration, owing to their intrinsic ability to differentiate into osteoblasts and secrete paracrine factors that can enhance bone regeneration [3, 4]. According to the minimal criteria to define human MSCs proposed by the International Society for Cellular Therapy (ISCT), among other features, MSCs should express surface markers CD73, CD90 and CD105 and lack of expression of haematopoietic markers CD45, CD34, CD19 and CD14 [5]. These minimal criteria were updated specifically for adipose tissue derived MSCs as they express CD34 [6]. Additional surface markers such as CD29, CD44 were also used to aid characterisation of MSCs from different tissues [7]. Over the past decade extensive research has endeavored to improve the therapeutic potency of MSCs through using phenotypically and functionally selected MSC sub-populations, with CD271 being a widely used marker for selecting functional MSC sub-populations [8]. Cell surface protein CD271 is recognized as a marker for MSC precursors in bone marrow and CD271-enriched bone marrow mononuclear cells (BM-MNCs) have been shown to yield more homogeneous MSC population with more potent tissue repair functions [9–12]. In standard 2D culture MSCs derived from CD271-enriched BM-MNCs have been shown to have higher proliferation rates and greater osteogenic differentiation potential compared to those from non-enriched counterparts [13]. Further study using 2D culture conditions evidenced a higher expression of osteoblast marker gene (*OPN*) in CD271-enriched BM-MSCs in comparison to non-enriched MSCs [14]. So far no study has been reported comparing heterogeneous and CD271-enriched MSC populations when cultured on 3D scaffold.

The implantation of a 3D scaffold into a bone defect is a promising tissue engineering approach to enhance osteogenic repair and regeneration by providing an optimal environment for cell attachment, proliferation and differentiation [15, 16]. A range of biomaterials has been used in combination with heterogeneous MSCs for bone engineering [17–20]. Apatite wollastonite glass ceramic (A-W) is an osteoconductive material which has been previously used clinically for load-bearing musculoskeletal repair [21]. It has the ability to bond to bone and to stimulate osteogenic differentiation in the surrounding cells [22]. Our previous work has led to the development of custom-built bone scaffold using heterogeneous MSCs and A-W glass-ceramics [23]. The biocompatibility of A-W scaffold and heterogeneous MSCs has been demonstrated through both in vitro dynamic culture and in vivo implantation in nude mice [24]. A-W is an attractive material for bone tissue engineering applications as it combines osteoconductivity with excellent mechanical properties: even when porous the mechanical properties can approach those of cortical bone [25]. This present study aimed to evaluate the attachment, proliferation and osteogenic potential of paired heterogeneous and CD271-enriched MSC populations when cultured on A-W 3D scaffolds.

## Results

### The frequency of CD271^high^CD45- cells before and after CD271 enrichment

**Fig. 1:**
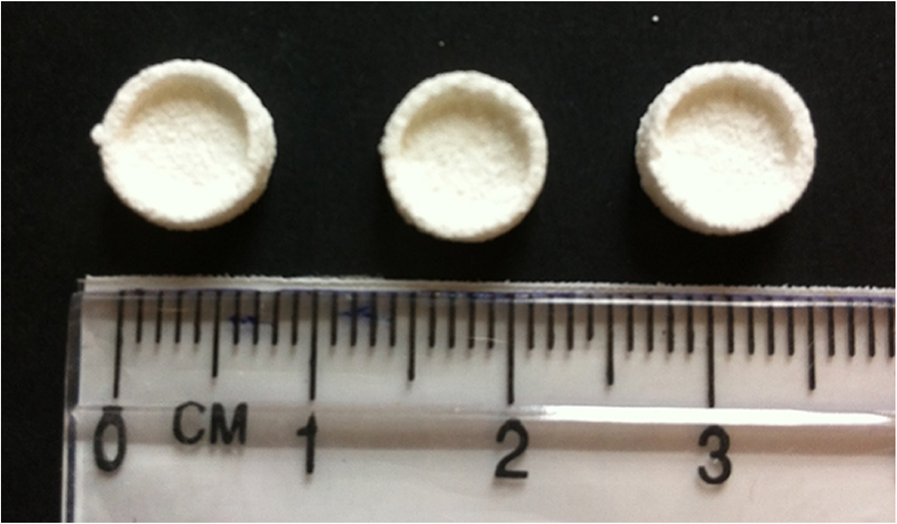
Frequency of CD271^high^CD45- cells before and after enrichment. The percentage (**a**) and representative flow cytometry dot plots (**b**) of CD271^high^CD45- cells in BM-MNCs before and after CD271-enrichment. Prior to the flow dot plot shown a live cell gate was applied. ***p < 0.0001 (paired *t*-test), Data shown are from 14 independent experiments

Prior to CD271 enrichment the average frequency of CD271^high^CD45- cells in the initial BM-MNCs was as low as 0.028% (±0.009). After CD271-enrichment the frequency of CD271^high^CD45- cells was elevated to 12.7% (±2.0). The enrichment resulted in an average of 1338-fold (±468) increase in the CD271^high^CD45- cells (Fig. 1a, b).

### PA-MSC and CD271-MSC show similar characteristics in 2D culture

**Fig. 2:**
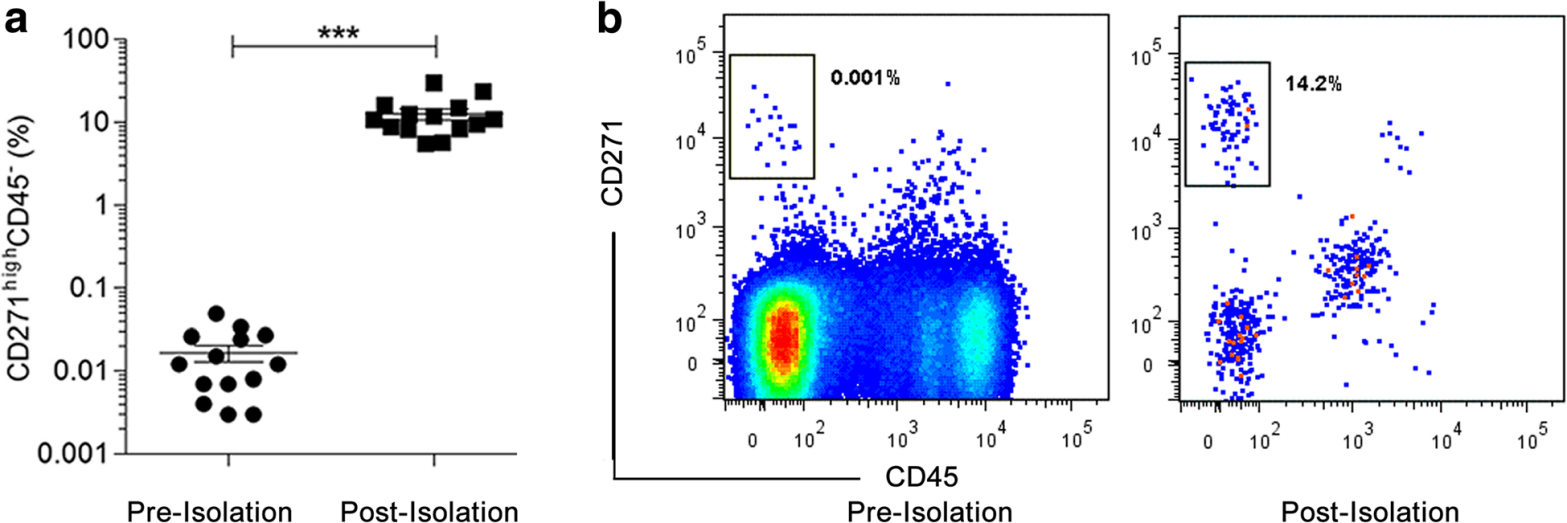
Characteristics of PA-MSC and CD271-MSC in 2D culture. Basic characteristics of paired PA-MSCs and CD271-MSCs were assessed during 2D in vitro expansion. **a** The cumulative population doublings of paired PA-MSC and CD271-MSC. **b**, **c** Representative phase contrast images showing the fibroblast-like morphology of PA-MSC (top row) and CD271-MSC (bottom row) respectively. Scale bars indicate 200 μm. **d**, **e** Representative images showing tri-lineage differentiation of PA-MSC and CD271-MSC respectively. From left to right: adipogenic differentiation (oil-red-O staining lipid vacuoles), osteogenic differentiation (ALP stain in blue and mineralization in black), chondrogenic differentiation (alcian blue staining glycosaminoglycans). Scale bars on all images indicate 200 μm. All images were taken on a NIKON spinning disk microscope. **f**, **g** Graphs showing the representative histogram and the percentage of positive cells for the phenotypic markers of MSCs. Error bars represent the SEM of 3 independent experiments

Paired MSCs were expanded in 2D culture up to 6 passages. The rate of proliferation was recorded during expansion. No difference was observed in the number of cumulative population doublings of PA-MSCs and CD271-MSCs (Fig. 2a). At passage 3 MSC populations were characterised using the standard criteria set by ISCT [5]. The results demonstrated that both PA-MSCs and CD271-MSCs displayed the standard MSC characteristics including plastic adherent, fibroblast-like morphology (Fig. 2b, c), ability to differentiate into osteogenic, chondrogenic and adipogenic lineages (Fig. 2d, e), positive expression of cell surface markers CD73, CD90, and CD105 and lack of expression of the hematopoietic lineage markers CD14, CD19, CD34, CD45 and HLA-DR (Fig. 2f,g). The characteristics expressed by CD271-MSCs were indistinguishable from those displayed by PA-MSCs.

### A-W scaffold seeded PA-MSC and CD271-MSC display different morphology

**Fig. 3:**
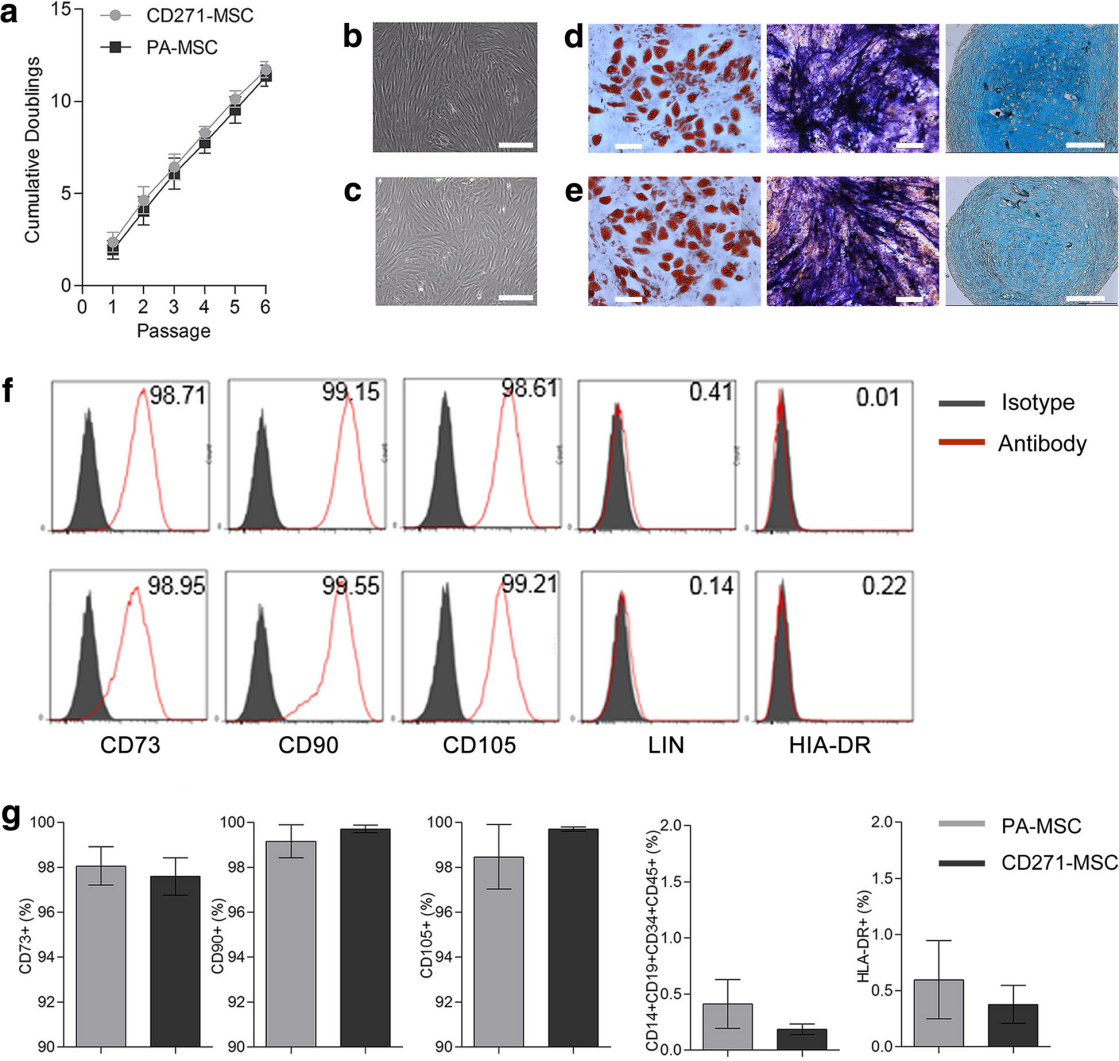
Morphology of A-W scaffold seeded PA-MSC and CD271-MSC. Representative images of scaffold seeded PA-MSC (**a**) and CD271-MSC (**b**) after 24 h culture in MSC expansion medium, in which the boxed areas were illustrated in higher magnification as (**c**) and (**d**) respectively. Phalloidin (red) stains the F-actin cytoskeleton showing elongated cell morphology. DAPI (blue) stains the nucleus and white/grey shows the surface of the scaffold. Images were taken with a Leica TCS SP2 UV AOBS MP scanning confocal microscope. Scale bars represent 150 μm (**a**) & (**b**) and 600 μm (**c**) & (**d**) respectively. Images are representative of 3 independent experiments. Analysis of morphology is shown with cell area (**e**) and circularity (**f**). Circularity is presented as frequency of occurrence in percentage. **p ≤ 0.01 (paired *t*-test). Error bars represent the SEM of 4 independent experiments

ImageJ analysis of Phalloidin and DAPI stained scaffolds (Fig. 3a, b) allowed for the quantification of cell size and shape. A-W scaffold seeded PA-MSCs were significantly larger and more elongated in shape compared to the CD271-MSCs 24 h after seeding (Fig. 3c) (*p* = 0.0055). Circularity, a measurement between 0 and 1, indicates how round a shape is, with 0 representing a straight line and 1 representing a perfect circle. PA-MSCs showed circularity values predominately at the linear end of the scale with 80% of PA-MSCs exhibiting a circularity range between 0.1 and 0.3 while over 70% of CD271-MSCs displayed middle range of circularity values between 0.3 and 0.6. (Fig. 3d), signifying a different level of attachment and interaction with the scaffold surface between PA-MSCs and CD271-MSCs.

### A-W scaffold seeded PA-MSC and CD271-MSC show different growth kinetic and varied scaffold integration

**Fig. 4:**
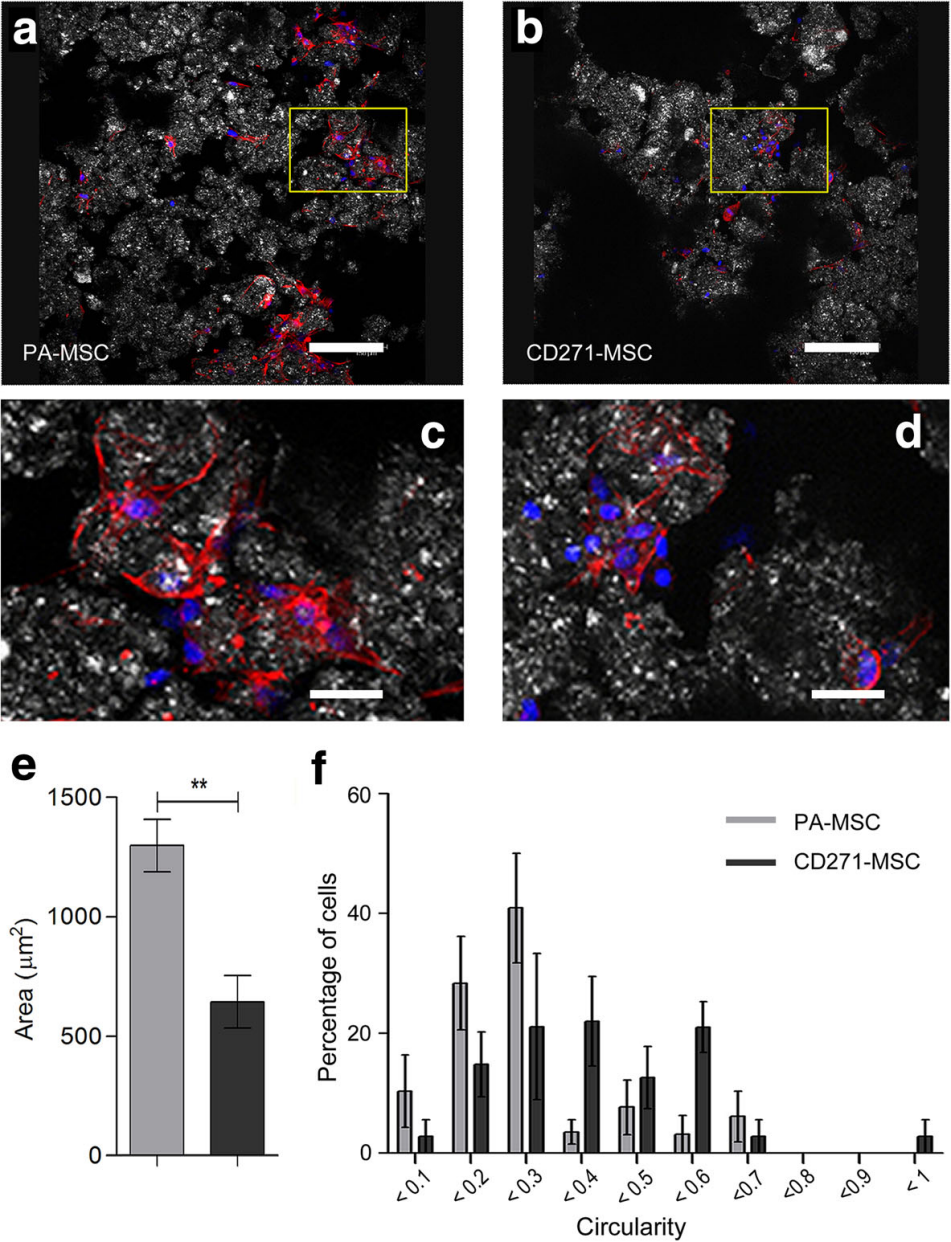
Growth kinetics of A-W scaffold seeded PA-MSC and CD271-MSC. **a** Graph showing the concentration of DNA obtained from MSC seeded scaffolds cultured in MSC expansion medium for 1, 3, 7 and 14 days. Day 0 value was obtained from unseeded cells. Error bars represent the SEM of 5 independent experiments. *** *p* ≤ 0.001 (two way paired ANOVA with Bonferroni post-test). **b-e** Scanning electron microscopy images showing MSC seeded scaffolds after 14 days of culture in MSC expansion medium. Scale bars represent 2 mm (**b**, **d**) and 500 μm (**c**, **e**). Images are representative of 3 independent experiments

The proliferation of A-W scaffold seeded MSCs was analysed using DNA content as a quantitative readout (Fig. 4a). Prior to being seeded on the scaffold (day 0) Both MSC populations showed similar base levels of DNA (76.7 ± 12.3 ng/ml and 75.9 ± 8.5 ng/ml respectively). At day 1, day 3 and day 7, there was a significant difference between the DNA content from scaffold seeded PA-MSCs and CD271-MSCs *(p ≤* 0.001). At day 1 and 3, this difference signified a larger PA-MSC population, but by day 7 this was reversed. Interestingly, between day 1 and day 3 both PA-MSCs and CD271-MSCs lost a similar amount of DNA (100–120 ng/ml). For the CD271-MSCs this represented a loss of 62% (±2%) of the population, while for the PA-MSCs this was only a loss of 37% (±2%). At day 7 the CD271-MSC population had increased, while the PA-MSC population had dropped further. Despite these differences, at day 14 the DNA concentration from the PA-MSC and CD271-MSC seeded scaffolds was almost identical, with 260.45 ng/ml (±7.5) and 263.2 ng/ml (±4.4) for PA-MSC and CD271-MSC respectively. Scanning electron microscopy images taken at day 14 showed that the scaffold seeded PA-MSCs had largely integrated into the scaffold hence less visible on the scaffold surface. On the contrary, the CD271-MSCs were highly visible on the surface of the scaffold (Fig. 4b-e), signifying poor integration into the scaffold compared to the PA-MSC population.

### A-W scaffold seeded PA-MSC and CD271-MSC show different osteogenic potential

**Fig. 5:**
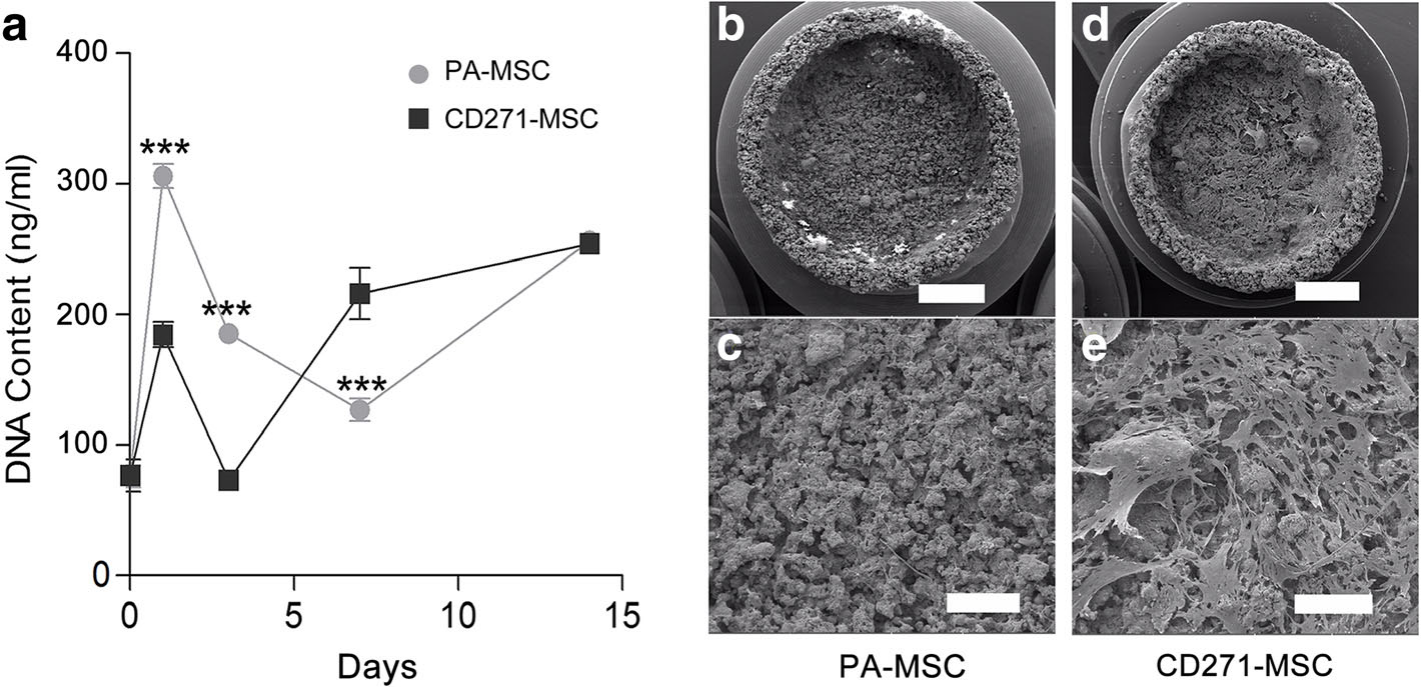
Osteogenic potential of A-W scaffold seeded PA-MSC and CD271-MSC. Scanning electron microscopy images (**a-d**) highlight areas of matrix deposition (*) and nodule formations (**<**) on MSC seeded scaffold after 14 days of culture in osteogenic induction medium. Scale bars represent 2 mm (**a**, **c**) and 50 μm (**b**, **d**). Images are representative of 3 independent experiments. Quantification of osteogenesis of paired MSCs is shown through ALP activity normalised to the DNA content (**e**). Error bars represent the SEM of 3 independent experiments. The osteogenic potential of non-cultured BM-MNCs seeded on A-W scaffolds, with or without CD271-enrichment, was presented as DNA quantification (**f**) and ALP activity (**g**). Error bars represent the SEM of 3 independent experiments. *p = 0.026 (paired *t*-test)

MSC seeded scaffolds were fixed after 14 days of osteogenic induction and analysed using high resolution scanning electron microscopy. Bone formation related structures were observed on the surface of the scaffold, such as fibrous ECM (web-like structure) and areas of mineralisation nodules as illustrated in Fig. 5a-d. Both structures were more prevalent on the PA-MSC seeded scaffolds compared to the CD271-MSC seeded scaffolds, indicative of a greater osteogenic potential of PA-MSCs on A-W scaffolds. The osteogenic potential of A-W scaffold seeded MSCs was also quantified using ALP activity as a readout, normalised to DNA content, after culturing the scaffolds in osteogenic media for 3, 7 and 14 days. A continued increase in ALP activity was observed in PA-MSCs across the 14 days of osteogenic induction (though not statistically significant) while little increase was observed in the ALP activity of CD271-MSCs (Fig. 5e).

To assess if the variation in osteogenic potential between the two A-W scaffold seeded MSC populations was due to culture expansion, freshly isolated uncultured BM-MNCs, with or without CD271-enrichment, were seeded onto A-W 3D scaffold and cultured in osteogenic media. Following 21 days of osteogenic induction the cell growth and osteogenic potential were analysed using DNA content and ALP activity as a quantitative readout respectively. The DNA content was significantly higher in non-enriched BM-MNCs compared to the CD271-enriched *(p =* 0.026) (Fig. 5f). Non-enriched BM-MNCs also showed an over 5-fold higher ALP activity (9621.8 μg/ml pNP/DNA μg/ml ± 5018.9) than that of CD271-enriched BM-MNCs (1781.6 μg/ml pNP/DNA μg/ml ± 601.8), although the statistical significance was not reached (Fig. 5g).

## Discussion

Our results indicate a higher osteogenic potential of A-W scaffold seeded heterogeneous PA-MSC compared to CD271-enriched MSC, despite previous evidence showing that purified uncultured CD271 positive BM-MNCs or 2D cultured CD271 enriched MSCs possess prominent osteogenic and Wnt signaling activity compared to their unselected or uncultured counterparts [14, 26, 27]. However, those results were generated from standard 2D plastic adhering cultures and the method used for CD271 enrichment in the reported study is different from the present study. To date, only one other study has combined cultured CD271-MSCs with a natural and synthetic polymer scaffold for osteogenic regeneration [28] by seeding rabbit culture expanded CD271-MSCs onto a 2D poly[ɛ-caprolactone]/thermoplastic zein-hydroxyapatite (PCL/TZ-HA) disk. This study reported a stronger osteogenic potential of CD271-MSCs than the PA-MSCs, but again was based on a 2D study. It is well documented that MSCs grown on 2D or 3D structural compositions have widely different properties [29–31], and here we demonstrate that, for our scaffold and cell populations, the differences between 2D and 3D culture can mean that results from 2D culture are a poor predictor of performance on a 3D scaffold.

Interestingly, compared to uncultured CD271-enriched BM-MNCs, we observed a higher DNA content and higher ALP activity in native BM-MNCs (unselected and uncultured) when seeded on the A-W scaffold. The unselected heterogeneous BM-MNCs may contain yet unknown accessory cells supporting MSC osteogenic potential. Most published work showing enhanced osteogenic gene expression of uncultured CD271 positive BM-MNCs used a highly purified CD271^high^CD45^−^ cell population isolated by FACS sorting [27, 32], whereas the present study utilised CD271-enriched rather than purified cells, which yield relatively lower frequency of CD271^high^CD45- cells but still produced an average of over 1300-fold increase in CD271^high^CD45- cells compared to non-enriched BM-MNCs.

The main observed differences between the two cell populations when seeded onto the scaffolds were:

- Different morphologies between the two A-W scaffold seeded MSC populations. PA-MSCs had elongated morphology and spread well on the scaffold whilst CD271-MSCs displayed a more round morphology and much sparse presence, suggesting poor attachment to the scaffold.
- Significantly different population expansion kinetics between the two populations (Fig. 4), with the PA-MSCs showing a much higher initial growth rate.
- The PA-MSCs fully integrated with the scaffold, the CD271-MSCs adhered to each other and formed a “plate-like” covering on the scaffold surface.
- The PA-MSCs showed a stronger propensity for osteogenic differentiation than CD271-MSCs when seeded on the A-W scaffolds.

The difference in scaffold attachment between the two MSC populations may have led to the population expansion, integration and differentiation differences. Previous research has shown that cells attached on stiff surfaces are more likely to develop an elongated morphology and undergo osteogenesis [33]. The connection between cell shape and differentiation is due to the force required by the cell to contract, and the strength with which the cells are attached to the material that they are seeded on [34]. Therefore, the strong and well-spread attachment of PA-MSCs to the A-W scaffold could have contributed to their higher osteogenic differentiation potential. The differences seen in attachment could be due to differences in adhesion molecule expression between the two populations. Such differences were not evident from standard MSC phenotyping using ISCT criteria, and are surprising given that the enriched cell population was derived from the non-enriched – there remain quite closely related cell populations. Improved understanding of adhesion mechanisms for cell population and 3D scaffold combinations would clearly help to enhance these types of tissue engineering approaches.

It is also possible that the different cell populations were affected through varying responses to stimuli from the scaffolds. Solutions containing the ionic products of bioactive glass ceramics are known to affect the proliferation rate in pre-osteoblast cells through alteration of the cell cycle and activation of apoptosis [35–38]. Variable susceptibility of PA-MSCs and CD271-MSCs to these stimuli could, at least partially, be an explanation for the differences in proliferation kinetics. Another suggestion [39] is that hydroxyapatite nanoparticles from calcium phosphate based materials can influence the epigenetic status of bone marrow derived stromal cells. As A-W forms a layer of hydroxyapatite on its surface during the process of ionic dissolution, it is possible that this mechanism played a part in the differences through selective targeting of specific cell sub-populations.

## Conclusions

This is the first study comparing the attachment, growth kinetics, and osteogenic potential of A-W scaffold seeded MSCs or BM-MNCs with or without CD271-enrichment. The key findings lead to the conclusion that an A-W scaffold seeded with unselected heterogeneous cells, cultured or uncultured, will show a more potent osteogenic potential than CD271-enriched cells, suggesting that enrichment of CD271-positive population is not beneficial for osteogenesis when the cells are cultured on an A-W scaffold, with or without culture expansion. Therefore, unselected heterogeneous cells (MSCs or BM-MNCs) are more promising for A-W scaffold based bone regeneration. In addition we conclude that care should be exercised in using 2D cell culture to indicate of likely performance of cell populations in a 3D scaffold based system, even where the cell populations are closely related – in this case one being an enriched population derived from the same starting population.

## Methods

### Cells and cell culture

Bone marrow-derived mononuclear cells (BM-MNC) were obtained using density gradient centrifugation over Lymphoprep (Axis-Sheld, Oslo, Norway). CD271^+^ BM-MNCs were isolated by immuno-magnetic positive selection using QuadroMACS system and clinical grade CD271 microbead kit (Miltenyi Biotec GmbH, Germany) following manufacturer’s instructions. The unselected and CD271-enriched BM-MNCs were then seeded into T25 culture flasks and 6 well culture plates respectively and cultured in good manufacturing practice (GMP) compliant MSC expansion medium containing 10% FCS (StemMACS, Miltenyi Biotec) at 37 °C in a 5% CO2 incubator. After 3 days, the non-adherent cells were discarded with the replacement of culture medium. The medium was changed twice weekly and cells were passaged to a new flask when the culture reached 80% confluence using standard Trypsin/EDTA (Sigma-Aldrich) treatment. The MSCs derived from BM-MNCs with or without CD271 enrichment were denoted as CD271-MSC and PA-MSC respectively. Each paired PA-MSC and CD271-MSC samples were generated from the same bone marrow donation, seeded at the same density (4 × 10^3^/cm^2^) and cultured/passaged under identical conditions. At each passage cell population doubling time was recorded. MSCs at passage 3 were used in all experiments throughout this work.

### Flow cytometry analysis

Flow cytometry analysis was performed to monitor MSC compliance with the phenotypic profile defined by the International Society for Cellular Therapy (ISTC) and to examine the frequency of CD271^high^CD45- cells in BM-MNCs before and after CD271 enrichment. The cells were stained with pre-optimised concentrations of antibodies or appropriate isotype controls for 30 min at 4 °C in FACS buffer, containing PBS with 2% FCS and 1 mM of endotoxin free ethylenediaminetetraacetic acid (EDTA). All antibodies were supplied by BD Biosciences including CD45-APC (HI30), CD271-PE (C40–1457), CD45-FITC (2D1), CD34-FITC (581), CD19-FITC (4G7), CD14_FITC (MφP9), HLA-DR-APC-H7 (L243), CD73-PE (AD2), CD90-PerCPCy5.5 (5E10), CD105-APC (266). Data were acquired on BD FACS Canto II cytometer and analysed using FlowJo software (Tree Star).

### Differentiation

In vitro differentiation was performed by culturing paired MSCs in adipogenic, osteogenic or chondrogenic media (all from Miltenyi Biotech), with media change twice a week. 2 × 10^5^ and 3 × 10^4^ cells were seeded in 6 well plates for adipogenesis and osteogenesis respectively. For chondrogenesis 2.5 × 10^5^ cells were pelleted and cultured in 15 ml polypropylene conical tubes with media change twice a week. Between 14 to 21 days of culture the cells were washed in PBS and fixed with 10% formalin followed by staining as previously described (Cuthbert et al.*,* 2015). Briefly, adipogenic cultures were stained for 10 min at room temperature with filtered 0.3% oil-red-O solution made with oil-red-O powder (Sigma) in undiluted isopropanol (Thermo-Fisher). After washing the cells were imaged to show lipid vacuoles within adipocytes. Chondrogenic pellets were paraffin embedded and sectioned (5 um) onto microscope slides. The slides were stained overnight at room temperature with 1% alcian blue (Sigma-Aldrich) solution in 0.1 N hydrogen chloride (HCL) (Thermo-Fisher), after sequential incubation in xylene (Thermo-Fisher) and decreasing concentrations of ethanol (Sigma), then imaged to show proteoglycan deposition. Osteogenic cultures were stained for both alkaline phosphatase (ALP) and mineralisation. ALP staining was performed overnight at 37 °C in a substrate containing Napthol-AS-phosphate (Sigma-Aldrich) and fast blue (Sigma-Aldrich) in 0.2 M Tris HCl solution (Thermo-Fisher, pH 9.0). After washing von Kossa staining was applied by adding 3% Silver Nitrate solution in dH_2_O and incubate for 1 h under direct light. Images of all stained cells were acquired using an inverted Nikon TIRF/Spinning Disk microscope.

### Scaffold seeding

**Fig. 6:**
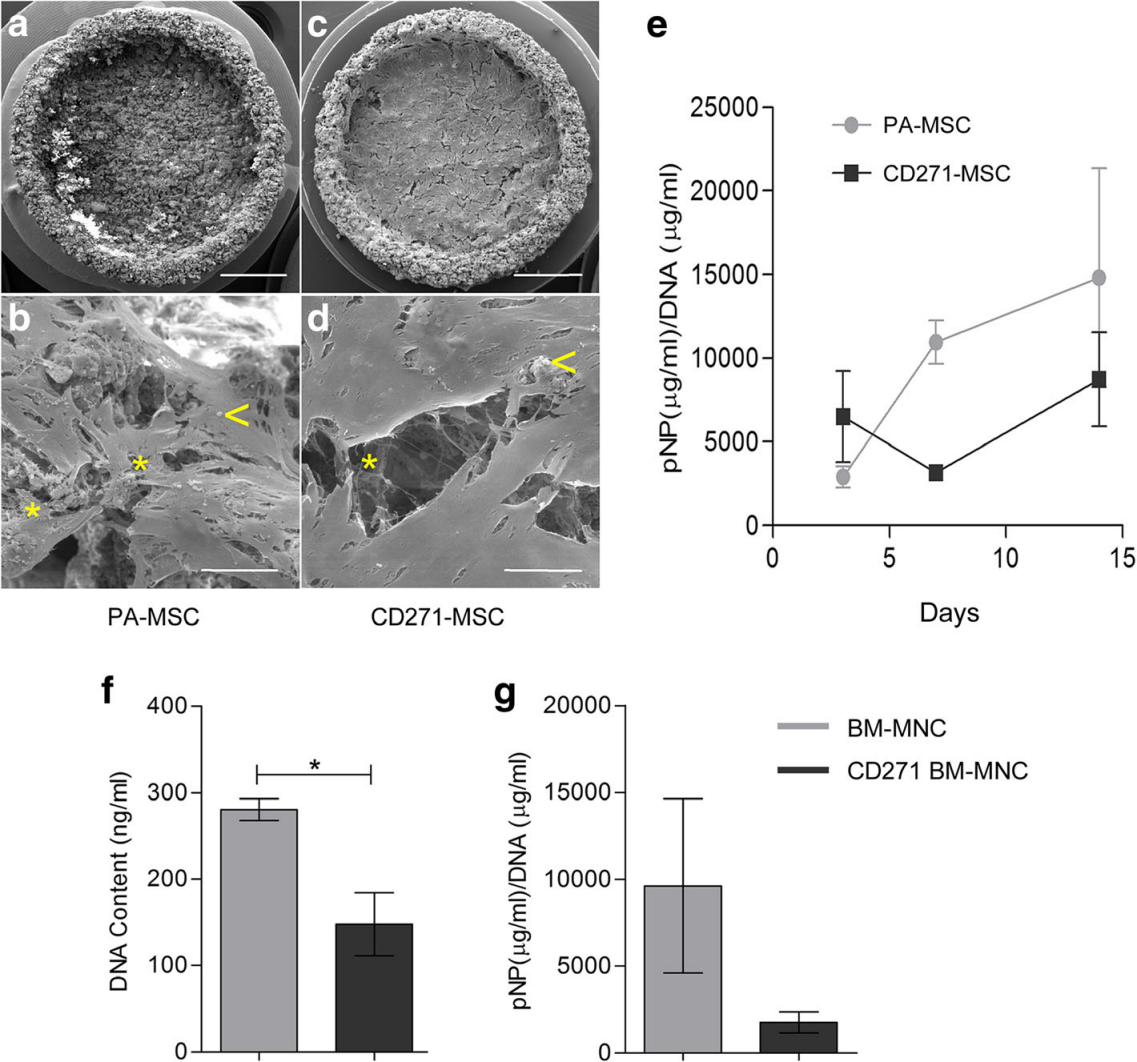
Overall shape and size of A-W scaffolds. A-W scaffolds were produced using the process described by Mancuso et al. (2017). Production involved the use of a Z Corp Z310 plus to print the 3D scaffolds from the A-W powder, followed by sintering in a furnace at 1150 °C to create a porous bowl shaped structure

A-W scaffolds were produced using the process described by Mancuso et al. [25]. Production involved the use of a Z Corp Z310 plus to print the 3D scaffolds from the A-W powder, followed by sintering in a furnace at 1150 °C to create a porous bowl shaped structure. The overall shape and size are illustrated in Fig. 6. Scaffolds were sterilized in an autoclave at 121 °C for 20 min, in accordance with previous work [23], soaked in PBS for at least 24 h then transferred to a 48 well plate and seeded with 5 × 10^4^ MSCs or freshly isolated non-expanded BM-MNCs in 20 μl StemMACS media. After 4–6 h incubation at 37 °C to allow cell adhesion, 1 ml of StemMACS media or osteogenic media was added to each well.

### Quantification of DNA content and ALP activity

DNA quantification was performed using a Quant-iT™ PicoGreen® assay (Life Tech) according to the manufactures recommendations. Briefly, the scaffold was incubated on ice for 20 min with 1 ml of lysis buffer (150 mM sodium chloride (Sigma-Aldrich), 1% Triton X-100 (Sigma-Aldrich), 0.1% sodium dodecyl sulphate (SDS) (Sigma-Aldrich) and 50 nM Tris (Thermo-Fisher, pH 8.0). The DNA standard was prepared at a range of concentrations from 10 ng/ml to 1000 ng/ml. Standards and cell lysates collected from the scaffolds were incubated in duplicate with PicoGreen reagent at room temperature in a 96 well plate for 5 min away from light then analysed on a Fluostar Omega plate reader (BMG Labtech). Quantification of ALP activity was performed using a phosphate substrate p-nitrophenylphosphate (pNPP) (Sigma-Aldrich) which produces p-nitrophenyl (pNP) when reacts with ALP. Known concentrations of pNP (Sigma-Aldrich) was used to generate a standard curve ranging from 2 μg/ml to 139 μg/ml. Standards and cell lysates collected from the scaffolds were incubated with substrate solution in a 96 well plate at 37 °C for 1 h. The reaction was stopped by 3 M NaOH (BDH Lab supplies) and the plate was read immediately at 405 nm on a Multiskan Ascent microplate reader (Termo-Fisher).

### Scanning confocal microscopy

Cell seeded scaffolds were cultured in expansion media for 24 h to allow for full attachment. Following routine wash and fixation the samples were stained with Phalloidin (Sigma Aldrich, 1:1000 in 0.1% DPBS/Tween) for 1 h at room temperature away from light. After further wash and mounting (Vectashield, with DAPI) the samples were imaged using a Leica TCS SP2 UV AOBS MP upright scanning confocal microscope with a water-dipping lens at × 20 magnification and 0.5 numerical aperture. To reflect the whole sample and minimise bias, images were acquired from at least 3 fields of each sample.

### Scanning electron microscopy

Cell seeded scaffolds were cultured in either StemMACS culture media or osteogenic induction media for 14 days then washed in PBS and fixed in 2% Glutaraldehyde in Sorenson’s Phosphate Buffer overnight. Scaffolds were then washed in Sorenson’s buffer and dehydrated in increasing concentrations of ethanol before being transported to Electron Microscopy Research Services (EMRS). After final dehydration with carbon dioxide in a Baltec Critical Point Dryer the scaffolds were mounted on aluminium stubs with Achesons Silver Dag, dried overnight then gold coated with 10 nm of gold in a Polaron Coating Unit. Scanning electron microscopy images were taken on a TESCAN VEGA SEM (Cambridge, UK) housed in Electron Microscopy Research Services at Newcastle University.

### Statistical analyses

All statistical analyses were performed with GraphPad Prism version 5 (GraphPad Software). Two-way paired ANOVA bonferroni post-test and paired *t*-test were used to determine statistical significance. Differences were considered statistically significant at a *p*-value of < 0.05.

## Data Availability

All relevant data generated and analysed during this study are included in this published article.

## Abbreviations

ALP: Alkaline phosphatase
A-W: Apatite-wollastonite
BM-MNC: Bone marrow mononuclear cells
CD271-MSC: CD271 enriched MSC
ISCT: International Society for Cellular Therapy
MSC: Mesenchymal stromal cells
PA-MSC: Heterogeneous plastic adherent MSC
pNP: P-nitrophenyl
