# Correction to: Osteogenic potential of heterogeneous and CD271-enriched mesenchymal stromal cells cultured on apatite-wollastonite 3D scaffolds

**DOI:** 10.1186/s42490-019-0033-9

**Published:** 2019-12-11

**Authors:** Sylvia Müller, Lyndsey Nicholson, Naif Al Harbi, Elena Mancuso, Elena Jones, Anne Dickinson, Xiao Nong Wang, Kenneth Dalgarno

**Affiliations:** 1 Institute of Cellular Medicine, Newcastle University, Newcastle upon Tyne, UK; 2 School of Engineering, Newcastle University, Newcastle upon Tyne, NE2 4HH UK; 3 Leeds Institute of Rheumatic and Musculoskeletal Medicine, University of Leeds, Leeds, UK

**Correction to: BMC Biomed Eng (2019) 1: 16.**

**Fig. 1:**
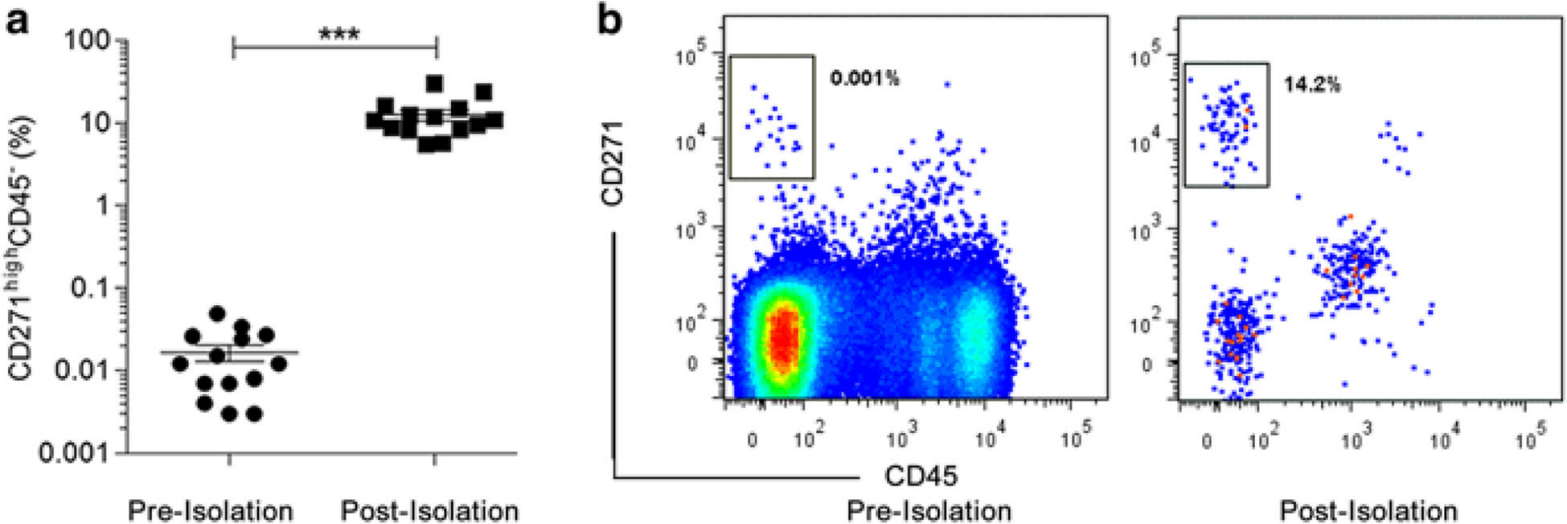
Frequency of CD271^high^CD45- cells before and after enrichment. The percentage (**a**) and representative flow cytometry dot plots (**b**) of CD271^high^CD45- cells in BM-MNCs before and after CD271-enrichment. Prior to the flow dot plot shown a live cell gate was applied. ***p < 0.0001 (paired *t*-test), Data shown are from 14 independent experiments

**Fig. 2:**
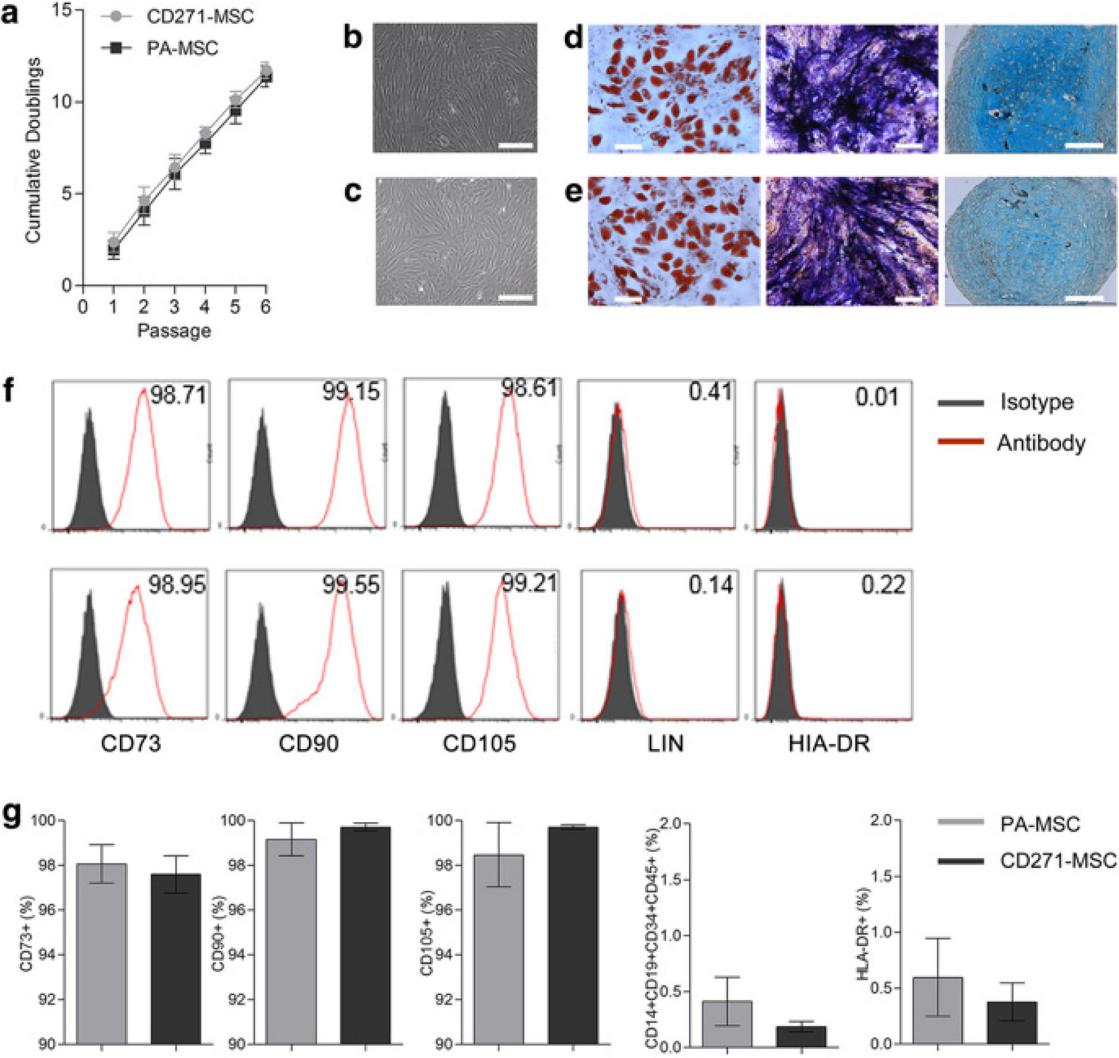
Characteristics of PA-MSC and CD271-MSC in 2D culture. Basic characteristics of paired PA-MSCs and CD271-MSCs were assessed during 2D in vitro expansion. **a** The cumulative population doublings of paired PA-MSC and CD271-MSC. **b**, **c** Representative phase contrast images showing the fibroblast-like morphology of PA-MSC (top row) and CD271-MSC (bottom row) respectively. Scale bars indicate 200 μm. **d**, **e** Representative images showing tri-lineage differentiation of PA-MSC and CD271-MSC respectively. From left to right: adipogenic differentiation (oil-red-O staining lipid vacuoles), osteogenic differentiation (ALP stain in blue and mineralization in black), chondrogenic differentiation (alcian blue staining glycosaminoglycans). Scale bars on all images indicate 200 μm. All images were taken on a NIKON spinning disk microscope. **f**, **g** Graphs showing the representative histogram and the percentage of positive cells for the phenotypic markers of MSCs. Error bars represent the SEM of 3 independent experiments

**Fig. 3:**
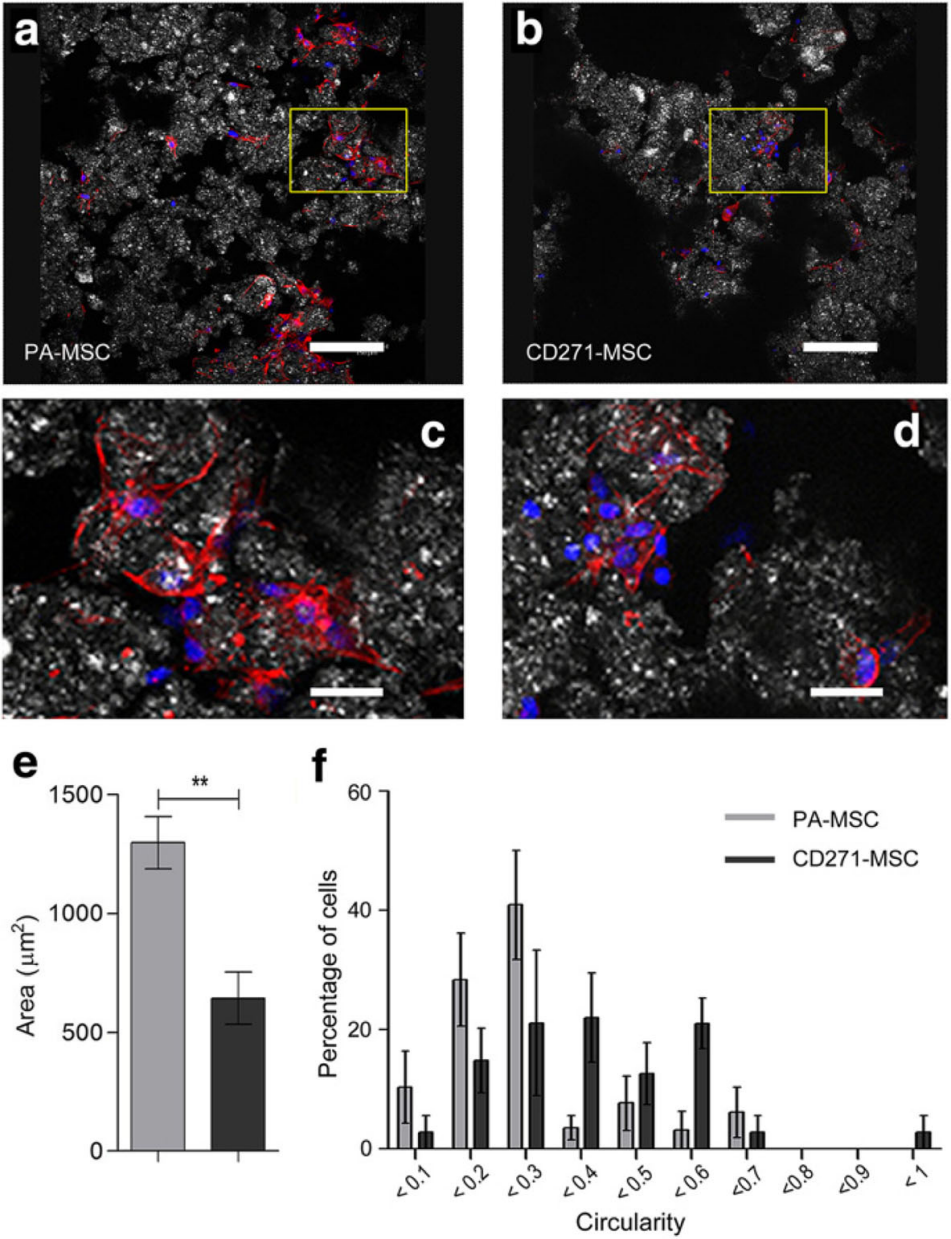
Morphology of A-W scaffold seeded PA-MSC and CD271-MSC. Representative images of scaffold seeded PA-MSC (**a**) and CD271-MSC (**b**) after 24 h culture in MSC expansion medium, in which the boxed areas were illustrated in higher magnification as (**c**) and (**d**) respectively. Phalloidin (red) stains the F-actin cytoskeleton showing elongated cell morphology. DAPI (blue) stains the nucleus and white/grey shows the surface of the scaffold. Images were taken with a Leica TCS SP2 UV AOBS MP scanning confocal microscope. Scale bars represent 150 μm (**a**) & (**b**) and 600 μm (**c**) & (**d**) respectively. Images are representative of 3 independent experiments. Analysis of morphology is shown with cell area (**e**) and circularity (**f**). Circularity is presented as frequency of occurrence in percentage. **p ≤ 0.01 (paired *t*-test). Error bars represent the SEM of 4 independent experiments

**Fig. 4:**
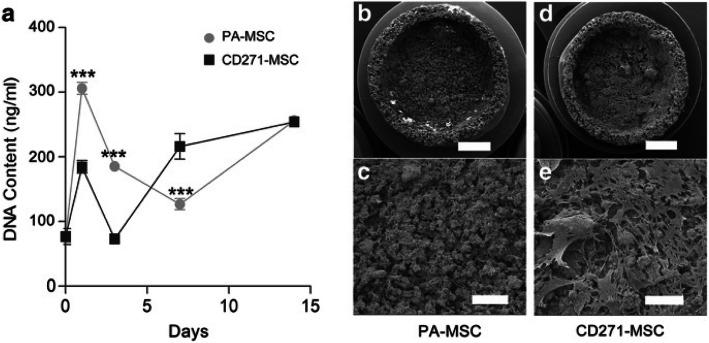
Growth kinetics of A-W scaffold seeded PA-MSC and CD271-MSC. **a** Graph showing the concentration of DNA obtained from MSC seeded scaffolds cultured in MSC expansion medium for 1, 3, 7 and 14 days. Day 0 value was obtained from unseeded cells. Error bars represent the SEM of 5 independent experiments. *** *p* ≤ 0.001 (two way paired ANOVA with Bonferroni post-test). **b-e** Scanning electron microscopy images showing MSC seeded scaffolds after 14 days of culture in MSC expansion medium. Scale bars represent 2 mm (**b**, **d**) and 500 μm (**c**, **e**). Images are representative of 3 independent experiments

**Fig. 5:**
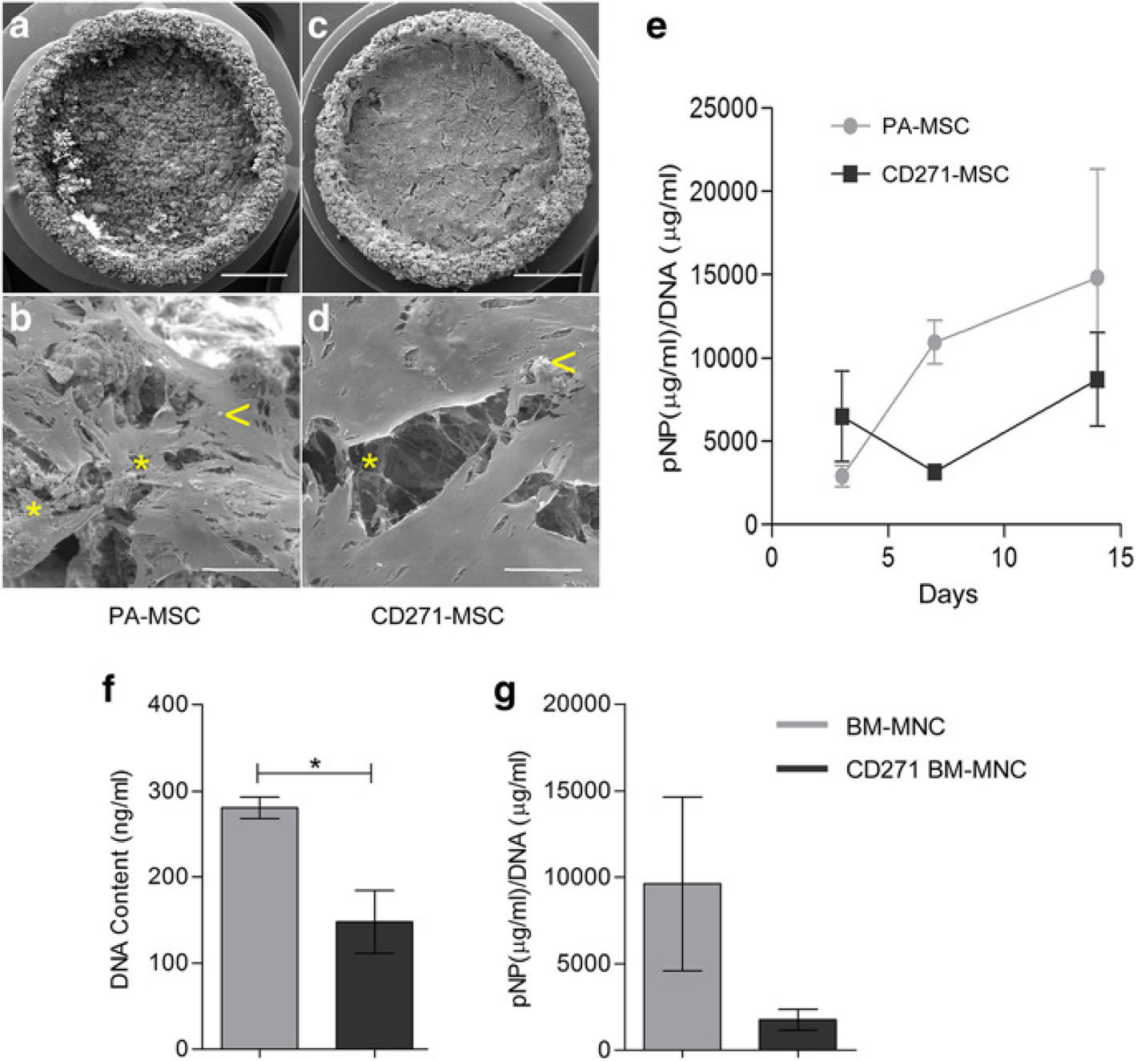
Osteogenic potential of A-W scaffold seeded PA-MSC and CD271-MSC. Scanning electron microscopy images (**a-d**) highlight areas of matrix deposition (*) and nodule formations (**<**) on MSC seeded scaffold after 14 days of culture in osteogenic induction medium. Scale bars represent 2 mm (**a**, **c**) and 50 μm (**b**, **d**). Images are representative of 3 independent experiments. Quantification of osteogenesis of paired MSCs is shown through ALP activity normalised to the DNA content (**e**). Error bars represent the SEM of 3 independent experiments. The osteogenic potential of non-cultured BM-MNCs seeded on A-W scaffolds, with or without CD271-enrichment, was presented as DNA quantification (**f**) and ALP activity (**g**). Error bars represent the SEM of 3 independent experiments. *p = 0.026 (paired *t*-test)

**Fig. 6:**
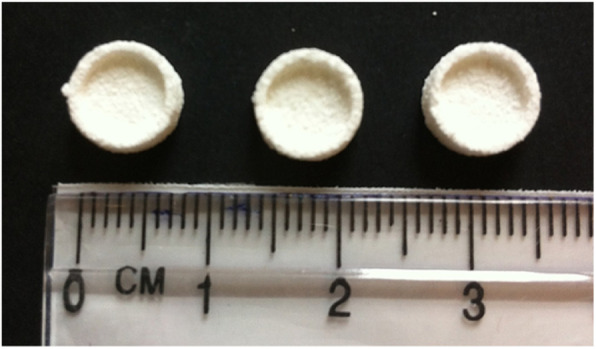
Overall shape and size of A-W scaffolds. A-W scaffolds were produced using the process described by Mancuso et al. (2017). Production involved the use of a Z Corp Z310 plus to print the 3D scaffolds from the A-W powder, followed by sintering in a furnace at 1150 °C to create a porous bowl shaped structure

**https://doi.org/10.1186/s42490-019-0015-y**

In the original publication of this article [1] the figures and captions were linked incorrectly. In this correction article the figures & captions are correctly published. The publisher apologizes to authors and readers for this error.
